# Crotoxin-Loaded Silica Nanoparticles: A Nanovenom Approach

**DOI:** 10.3390/pharmaceutics17070879

**Published:** 2025-07-04

**Authors:** Florencia Silvina Conti, Exequiel Giorgi, Laura Montaldo, Juan Pablo Rodríguez, Mauricio Cesar De Marzi, Federico Gastón Baudou

**Affiliations:** 1 Grupo de Investigaciones Básicas y Aplicadas en Inmunología y Bioactivos (GIBAIB, INEDES-CONICET), Departamento de Ciencias Básicas, Universidad Nacional de Luján (UNLu), Luján C6700, Argentina; fconti@unlu.edu.ar (F.S.C.); egiorgi@mail.unlu.edu.ar (E.G.); laura.montaldo24@gmail.com (L.M.); federicobaudou@gmail.com (F.G.B.); 2 FOCIS Center of Excellence—Vaccines and Immuno Therapies Against Infections and Cancers (VITIC), Menomonee Falls, WI 53051, USA; 3 Laboratorio de Investigaciones Bioquímicas, Facultad de Medicina (LIBIM), Instituto de Química Básica y Aplicada del Nordeste Argentino (IQUIBA-NEA), Universidad Nacional del Nordeste, Consejo Nacional de Investigaciones Científicas y Técnicas (UNNE-CONICET), Corrientes W3400, Argentina; rodriguezcasco@gmail.com; 4 Laboratorio de Glicomedicina, Instituto de Biología y Medicina Experimental, Consejo Nacional de Investigaciones Científicas y Técnicas (IBYME-CONICET), Buenos Aires C1428, Argentina

**Keywords:** SiNPs, snake venom, PLA_2_, serotherapy, nanovenoms, adjuvants

## Abstract

**Background**: Ophidism is a globally neglected health problem. In Argentina, *Crotalus durissus terrificus* (*C.d.t*., South American rattlesnake) is one of the species of greatest medical importance since its venom contains mainly crotoxin (CTX), a potent enzyme–toxin with PLA_2_ activity, which is responsible for its high lethality. **Objective**: In this work, we aimed to generate nanovenoms (NVs), complexes formed by CTX adsorbed onto 150 nm silica nanoparticles (SiNPs), and to study their physicochemical, biological, and immunomodulatory activities for potential use as adjuvants (ADJs) in antivenom (AV) production. **Methods**: CTX was isolated and corroborated by SDS-PAGE. Then, CTX was adsorbed on the synthetized Stöber SiNPs’ surfaces, forming a monolayer and retaining its biological activity (as observed by the MTT cell proliferation assay using the THP-1 cell line). **Results**: Immunomodulatory activity revealed a high pro-inflammatory (IL-1β) response induced by SiNPs followed by NVs. In the case of the anti-inflammatory response, NVs presented significant differences for TGF-β only after cell activation with LPS. No significant differences were observed in IL-10 levels. **Conclusions**: Thus, these results suggest that NVs together with SiNPs could increase immunogenicity and enhance immune response, turning them into potential tools for the generation of new antivenoms.

## 1. Introduction

Ophidism refers to injuries caused by venomous snake bites. This health issue is globally neglected and has been declared an area of great interest by the World Health Organization [1], mainly due to the clinical manifestations of envenomation caused by these animals [2,3,4]. More than 5 million snakebite incidents occur annually worldwide, involving approximately 300 venomous snake species; of these cases, over half a million result in permanent sequelae, such as limb amputations, and about 150,000 lead to fatalities [5,6,7]. In Argentina, snakes belonging to the family Viperidae are responsible for most snakebite incidents [8]. The genus *Bothrops* (yararás) is responsible for more than 90% of accidents, while the remainder are almost entirely caused by *Crotalus durissus terrificus* (*C.d.t.*), commonly known as the South American rattlesnake.

Ophidic venoms are modified salivary secretions, produced by specialized exocrine glands [9] exhibiting enzymatic activity similar to that of the exocrine pancreas [10]. These venoms consist of more than 90% proteins by weight, containing small peptides and toxins [11]. Some of these enzymatic proteins include metalloproteinases, serine proteases, L-amino acid oxidases (LAAOs), and phospholipases A_2_ (PLA_2_), among others [12]. In particular, *C.d.t.* venom is primarily composed of crotoxin (CTX), which constitutes over 60% of the venom’s total weight and is largely responsible for its high lethality [13]. In addition, *C.d.t.* venom contains thrombin-like enzyme (TLE), convulxin (CVX, a C-type lectin-like protein or CTL), and, in some specimens, crotamine (CTM), all of which significantly contribute to the neurotoxic and myotoxic effects of envenomation [14]. The CTX complex is a heterodimeric protein composed of a basic and toxic subunit called CTX B, with PLA_2_ activity, and an acidic subunit called crotapotin, which acts as a chaperone for CTX B [15]. This complex hydrolyzes phospholipids and affects peripheral neurotransmission, acting at the presynaptic level in neurons at the myoneural junction, altering their permeability properties and interactions with membrane-bound proteins [16]. Therefore, the signs of crotalic envenomation are mainly neurotoxic and myotoxic [17,18]. This complex can be easily purified, reproducing all the pharmacological effects of CTX, and can be used as an isolated component for the manufacture of specific antivenoms (AVs) or in medical applications [19,20].

The generation of AVs is a costly and time-consuming process that involves immunization protocols in animals. These animals are inoculated with increasing doses of the target venom and/or its isolated toxins, combined with adjuvants (ADJs) to enhance their immune response [21]. However, using large quantities of toxins and traditional ADJs (such as Freund’s complete and incomplete ADJs) in repeated doses can harm the animals. This damage could be mitigated by designing alternative protocols that include other types of ADJs and/or reduced the quantities of venoms or toxins [22,23].

Nanoparticles (NPs) are currently being studied for multiple purposes and have generated great interest due to their potential therapeutic applications [24,25,26]. A variety of materials, including gold, iron, and chitosan, are used for NP synthesis. Magnetic and silica NPs are of particular interest because their surfaces can be chemically modified to improve interactions with biological molecules [27]. These nanometer-sized compounds have a wide range of applications, including their use as carriers of molecules involved in the immune response or the modulation of the immune response by producing immunostimulation or immunosuppression processes [28,29,30].

In this study, we propose the use of NPs combined with snake venoms or their isolated components, such as CTX, to improve AV production in the future. This approach could lead to more effective and economical AV while minimizing harm to the animals employed in the process. For this purpose, we investigated the physicochemical, biological, and immunomodulatory activities of nanovenoms (NVs), which are complexes formed by CTX isolated from *C.d.t.* venom and 150 nm silica NPs (SiNPs) with both positive and negative net charges.

## 2. Materials and Methods

### 2.1. Venom (V)

*C.d.t.* venom was obtained from adult specimens of both sexes from the Centro de Conservación Aguará, Paso de la Patria, Corrientes (Argentina), and from the Instituto Carlos G. Malbrán, Buenos Aires, CABA (Argentina). Snakes were milked and their secretion was homogenized, lyophilized, and kept frozen at −20 °C. The small amount of insoluble material was centrifuged, and the clear supernatant (SN) was used for studies. The protein content was calculated using the Bradford method.

### 2.2. Isolation of CTX from C.d.t. Venom

Using fast protein liquid chromatography (FPLC) (GE Healthcare, Chicago, IL, USA, Superdex 75), fractions containing CTX were isolated from whole *C.d.t.* venom using 0.1 M NH_4_HCO_3_, pH = 8, as a running buffer. After that, the fractions were dialyzed for 24 h against 10 mM PBS buffer at 4 °C under stirring, then lyophilized and redissolved in 10 mM PBS buffer matching the dialyzed volume. Its purity was corroborated by SDS-PAGE and the performance was calculated by ImageJ (v.1.53) analysis. The isolated CTX (0.6 mg/mL) was kept frozen (−20 °C) until further use.

### 2.3. Synthesis of Silica Nanoparticles (SiNPs)

Solid 150 nm silica nanoparticles (SiNPs) were synthesized by the sol–gel technique according to the Stöber method [31]. For the synthesis, 6 mL of 98% tetraethyl orthosilicate (TEOS) was added drop by drop to a solution of 79 mL of ultrapure water, 118 mL of absolute ethanol, and 12 mL of 25% ammonium hydroxide (NH_4_OH) and stirred overnight (ON) at room temperature (RT). This process resulted in NPs with a net negative charge (SiOHNPs). Finally, a portion of these were subjected to surface modifications with the addition of APTES (3-aminopropyltriethoxysilane), producing SiNPs with net positive charge (SiNH_2_NPs) [32,33]. The SiNPs were washed with miliQ H_2_O and then dried to evaporate the water.

### 2.4. Immobilization of CTX on SiNPs (Synthesis of NVs) and Adsorption Studies

For this purpose, 10 mg of the SiNPs obtained from both charges (SiNH_2_NPs/SiOHNPs) was incubated in the presence of CTX (1 mg/mL) (final volume of 1 mL in 10 mM PBS buffer) under stirring for 3 h at RT. Subsequently, NPs were centrifuged for 10 min at 6700× *g*, the supernatant (SN) was collected, and the pellet was resuspended in PBS buffer. This last step was repeated at different times (30 min, 1, and 2 h) at RT in order to study the detachment of CTX under these conditions (stability study of the complex). Then, the SNs were stored to determine the concentration of CTX, measuring desorption by spectrophotometry (abs 280 nm). The concentration of CTX adsorbed onto the SiNPs was calculated as the difference between the initial CTX concentrations used and those measured in the SN. The adsorption capacity was also calculated as the amount of CTX adsorbed relative to the SiNP concentration used for NV synthesis. To better understand the characteristics of the CTX adsorption process on the surface of the SiNPs, adsorption isotherms were created, and their adjustment to Langmuir and Freundlich models was calculated [33].

### 2.5. Physicochemical Characterization of SiNPs and NVs

The physicochemical characterization of the synthesized SiNPs, as well as the NV complexes, was carried out by infrared spectroscopy (Nicolet iS 50 FT-IR spectrophotometer (Thermo Fisher Scientific, Waltham, MA, USA), with a KBr beamsplitter (Omega, Hopewell Junction, NY, USA)) to evaluate the presence of different functional groups and chemical bonds characteristic of each compound (Si-O-Si bonds and silanol groups [Si-OH] in SiNPs and S-S bonds in CTX). Spectra were obtained with a resolution of 2 cm^−1^ using a DTGS detector. The samples were measured using the attenuated total reflectance (ATR) technique. For this, 150 μL of the samples were dried under a nitrogen flow and the powder was then placed on the attenuated total reflection component of the spectrometer and the spectra were recorded. Also, the spectrometer was used to corroborate the adsorption of CTX over SiNPs (NVs).

In addition, the adsorbed capacity of CTX on the SiNPs (AC) and the load capacity of NVs (LC) were, respectively, calculated as

$$\mathrm{AC}=\frac{CTX(\frac{mg}{ml})}{non-adsorbed\;CTX(\frac{mg}{ml})}\;\;\;\;\mathrm{LC}=\frac{CTX(\mu g)}{SiNPS(mg)}$$

where, for AC, CTX (mg/mL) refers to the protein that was successfully adsorbed onto the SiNPs and non-adsorbed CTX (mg/mL) corresponds to the protein that was not adsorbed onto the surface of SiNPs. On the other hand, for LC, CTX (µg) refers to the amount of protein adsorbed onto the NPs and SiNPs (mg) correspond to the amount of NPs used for the synthesis of NVs.

The morphology of the SiNPs and NVs was examined with a transmission electron microscope (TEM Zeiss EM109 T at 200 keV, Zeiss, Jena, Germany). For the analysis, SiNP and NV suspensions were diluted 1:20 in miliQ H_2_O, and 10 μL was deposited into carried grids (S147−4, Plano GmbH, Wetzlar, Germany) and allowed to dry under ambient conditions before imaging. The zeta potential (ζ) and the hydrodynamic size were determined by dynamic light scattering (DLS Litesizer 500 Anton Paar, Graz, Austria). For this purpose, SiNPs were suspended in miliQ H_2_O (0.1 mg/mL); suspensions were sonicated in an ultrasonic bath for 10 min and then measured.

### 2.6. Analysis of CTX Desorption from NVs by SDS-PAGE

For this study, 30 μL of whole venom of *C.d.t.* (1 mg/mL), CTX (0.60 mg/mL), and CTX detached from NVs (SN+ (0.67 mg/mL) corresponding to SiNH_2_NPs and SN− (0.51 mg/mL) corresponding to SiOHNPs were resolved on a 14% SDS-PAGE. The gel was stained with 0.1% Coomassie blue R-250 (in 40% methanol and 12% acetic acid) overnight and de-stained with 10% acetic acid (with replacement) for 8 h.

### 2.7. Study of Biological Activity of SiNPs and NVs

#### 2.7.1. PLA_2_ Indirect Hemolytic Activity

For this assay, 10 µL of whole venom (1 mg/mL in 10 mM PBS), CTX (0.6 mg/mL in 10 mM PBS), SiNH_2_NPs (0.60 mg), SiOHNPs (0.60 mg), and NVs of both charges (resuspended in PBS, 0.67 mg CTX/10 mg SiNH_2_NPs and 0.51 mg CTX/10 mg SiOHNPs) were added to wells made in blood agar (0.7 g agarose, with 70 mL 10 mM PBS, 0.8 mL washed erythrocytes, 0.8 mL egg yolk—1:3 H_2_O—0.03 mL 0.25 M CaCl_2_). The plates were incubated in a humidified chamber for 24 h at 37 °C, and the diameters of the hemolysis halos were subsequently measured [34].

#### 2.7.2. Cell Proliferation Assay (MTT)

THP-1 cells were cultured using RPMI 1640 medium supplemented with 10% FBS, 2 mM glutamine, 1 mM pyruvate, 100 U penicillin/mL, and 100 μg streptomycin/mL (complete medium) (37 °C with 5% CO_2_ and a humidified incubator). Viable cells were counted in the Neubauer chamber after staining with Trypan Blue. Briefly, THP-1 cells (1 × 10^5^ cells/mL) were grown in 24-well plates in the presence of complete medium (control), whole venom (10 μg/mL), CTX (10 μg/mL), NV+ (10 µg/mL CTX in 0.17 mg SiNH_2_NPs), NV− (10 µg/mL CTX in 0.21 mg SiOHNPs) for 48 H, both NVs calculated by AC, and the different SiNPs (0.6 mg/mL) in triplicate (final volume: 1 mL/well). The tetrazolium assay (MTT) [35] was used to assess cell proliferation. For this, cells were centrifuged, resuspended in MTT solution (5 mg/mL in PBS), and incubated at 37 °C in a humidified environment for 1 h. Then, 200 μL of EtOH was added and centrifuged for 5 min at 5000× *g*. Finally, the absorbance of the solution was measured at 620 nm using a spectrophotometer.

#### 2.7.3. Immunomodulation by NVs on THP-1 Cells

Cytokine secretion (IL-1β, IL-10, and TGF-β) was measured in THP-1 SN and in THP-1 SN previously stimulated with LPS (50 ng/mL) by enzyme-linked immunosorbent assay (ELISA) kits, following the manufacturer’s instructions (BD Biosciences, Franklin Lakes, NJ, USA). Absorbance was measured at 450 nm on a microplate reader (FLUOstar Omega, BMG Labtech, Ortenberg, Germany), and the cytokine concentration was calculated from standard curves, expressed in pg/mL.

Nitrite production was also analyzed in supernatants (SNs) using Griess reagent, with different concentrations of sodium nitrite as the standard control. Briefly, 50 µL of each commercial reagent (sulfanilic acid and dimethyl-α naphthylamine) was mixed with 100 µL of the sample or standard control, incubated for 15 min, and then read at 560 nm (FLUOstar Omega).

## 3. Results

### 3.1. Characterization of SiNPs

**Figure 1:**
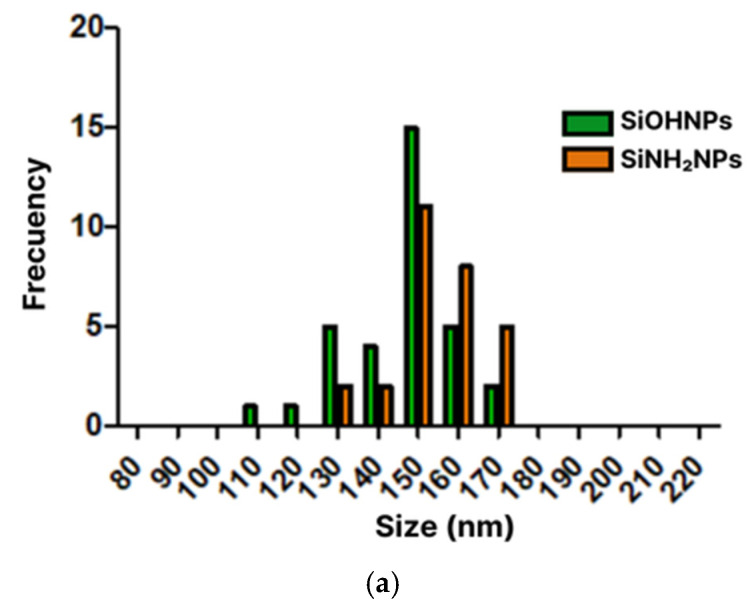

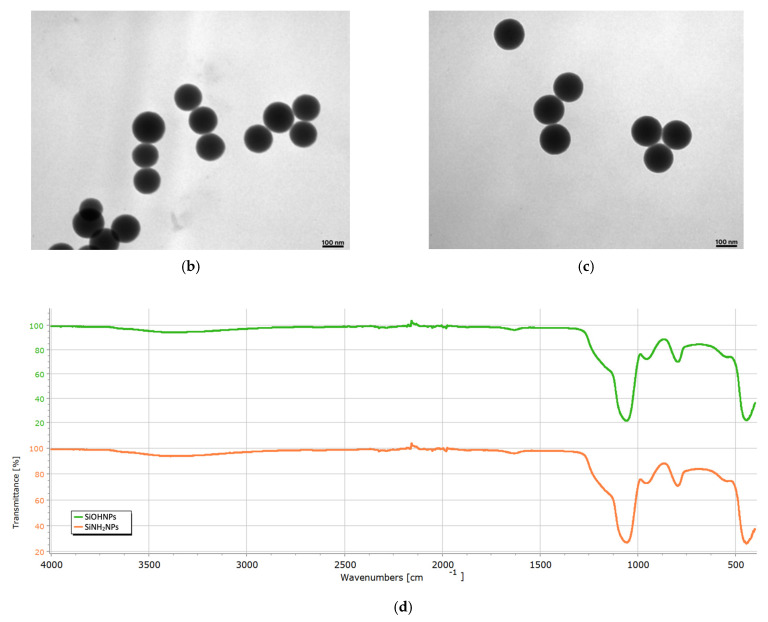
Characterization of SiNPs. (**a**). Dynamic light scattering (DLS). Histogram of the diameters of the SiNPs obtained by DLS. Transmission Electron Microscopy (TEM) micrographs. Bare silica morphologies of SiOHNPs (**b**) and SiNH_2_NPs (**c**) obtained according to the Stöber method. Scale bar: 100 nm. (**d**). FTIR: infrared spectra of SiOHNPs (green) and SiNH_2_NPs (orange).

The mean hydrodynamic diameters of the SiNPs, obtained by DLS, were 146 ± 12 nm for SiOHNPs and 154 ± 10 nm for SiNH_2_NPs (Figure 1a), and the polydispersity index (PDI) was 0.24 and 0.29, respectively. The zeta potential (ζ) was −28 mV and 15 mV for the negative and positive SiNPs, respectively. Micrographs of both types of SiNPs were obtained using TEM, showing the circular and uniform morphology of the synthesized material (Figure 1b,c). The size was corroborated by measuring the diameters of the SiNPs in the micrographs using ImageJ, yielding 144 ± 7 nm for SiOHNPs and 145 ± 3 nm for SiNH_2_NPs. The presence of characteristic functional groups was also evaluated by Fourier-transform infrared spectroscopy (FT-IR). Absorption bands corresponding to the Si–O–Si and Si–O–H bonds were observed at 1100 cm^−1^ and 960 cm^−1^, respectively, for both SiNH_2_NPs and SiOHNPs (Figure 1d).

### 3.2. Isolation and Quantification of CTX

**Figure 2:**
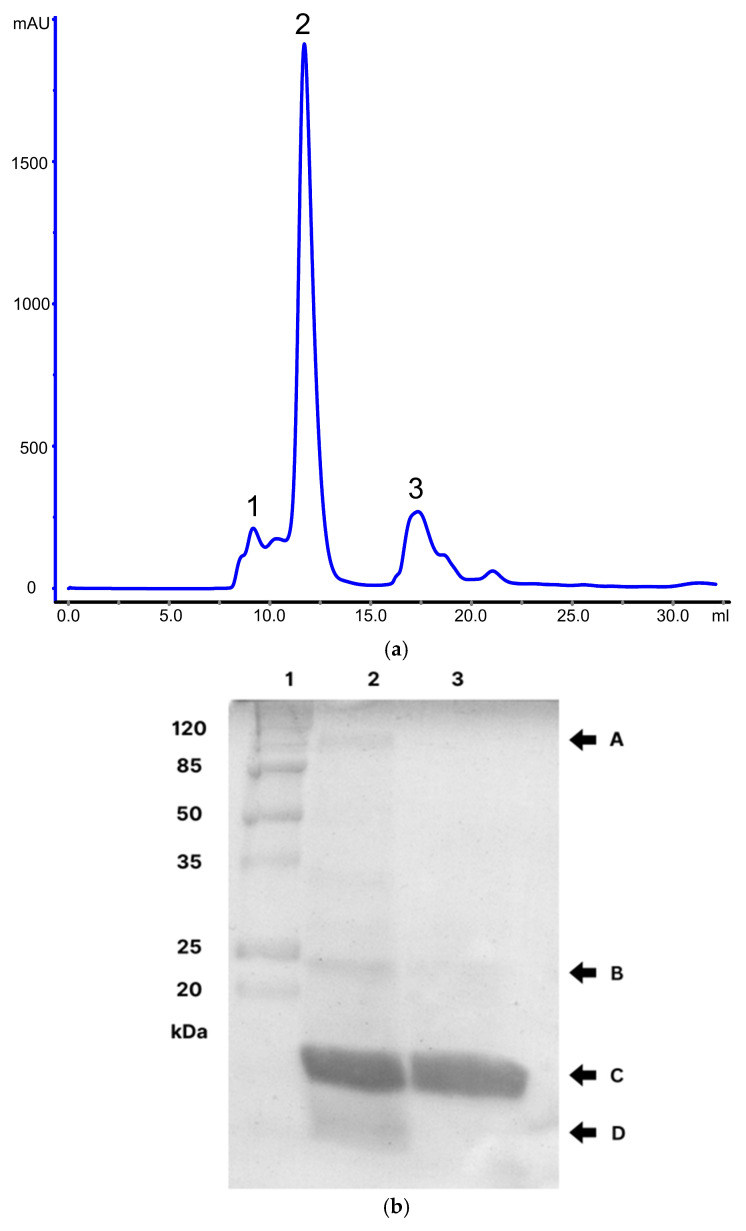
Isolation and quantification of purified CTX. (**a**). Elution plot. Elution chromatographic profile resulting from FPLC. The elution peaks refer to convulxin (1), crotoxin (2), and crotamine (3). (**b**). SDS-Page: polyacrylamide gel electrophoresis (14%) of whole *C.d.t.* venom and CTX. Molecular mass marker (1), *C.d.t.* venom (2), and isolated CTX (3). Black arrows indicate the venom proteins: CVX (A), LAAO (B), CTX (C), and CTM (D).

CTX was isolated from the whole *C.d.t.* venom using FPLC. The elution plot (Figure 2a) shows three distinct peaks. The first one (elution volume: 8 mL) was attributed to CVX (1). The largest peak (11 mL) corresponded to CTX (2). The third peak (16 mL) was identified as CTM (3), a small basic myotoxin (~5 kDa) [14,36]. The isolated CTX was quantified using spectrophotometry (280 nm), yielding a concentration of 0.6 mg/mL. Its purity percentage was confirmed by SDS-PAGE (Figure 2b) and ImageJ analysis, obtaining 97.18%.

### 3.3. Immobilization and Characterization of CTX Adsorbed to SiNPs (NVs)

**Figure 3:**
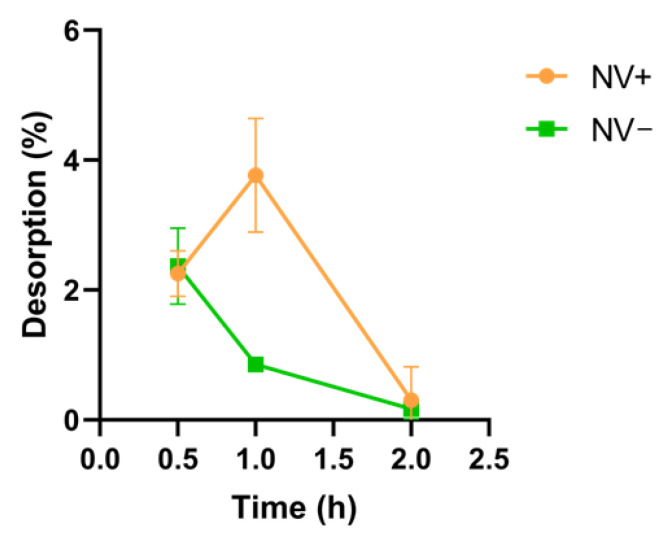
Desorption of CTX. Stability over time of CTX adsorbed onto NVs+/−. The graph shows the percentage of desorption (mean ± SD) of this protein in both types of NVs at different times (30 min, 1 and 2 h).

The concentration of adsorbed CTX was determined using UV–visible spectrometry by measuring absorbance at 280 nm. For this purpose, the difference between the concentration initially used for CTX immobilization on the surface of both types of SiNPs and the non-adsorbed CTX remaining on the supernatant (SN+/SN−) after incubation was calculated (mean ± S.D. *n* = 3). The procedure was repeated after 30 min, 1 h, and 2 h of incubation at RT. This allowed for the quantification of CTX desorption from the NVs over time. The LC was calculated, resulting in 66.8 µg of CTX/mg SiNPs for SiNH_2_NPs and 51.4 µg of CTX/mg SiNPs for SiOHNPs, with both adsorption capacities being greater than 50%, taking the initial concentration of CTX (1 mg/mL) as 100%. These results were obtained by measuring the amount of CTX desorbed at different times after NV synthesis. At time 0, CTX desorption was below 50% for both NV+ and NV− (33.2% ± 7.09 and 48.6% ± 6.16, respectively). After 30 min, the desorption values significantly decreased to 2.25% ± 0.35 for NV+ and 2.37% ± 0.59 for NV−, like those obtained at the 1 h mark (3.77% ± 0.87 in NV+, 0.85% ± 0.07 in NV−). After 2 h, desorption was negligible. The adsorption of CTX was higher for SiNH_2_NPs (0.67 mg/mL) compared to the SiOHNPs (0.51 mg/mL) (Figure 3)**.**

**Figure 4:**
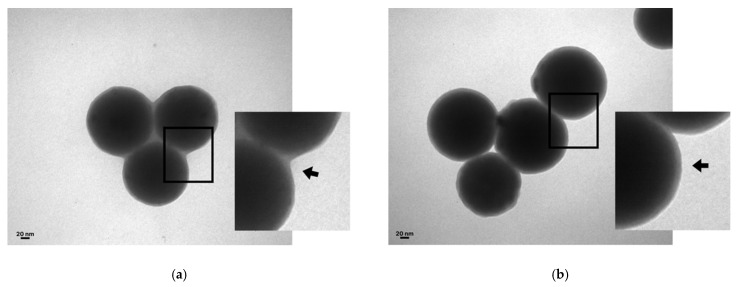

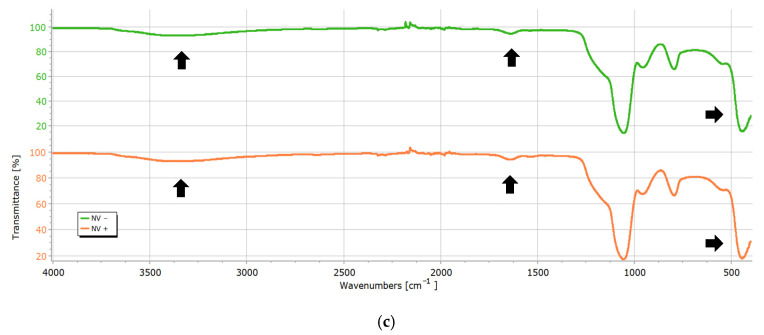
TEM micrograph. Micrographs of NV− (**a**) and NV+ (**b**) at 150 nm. The adsorbed CTX is indicated by a black arrow. Scale bar: 20 nm. (**c**). FTIR: infrared spectra of NV− (green) and NV+ (orange). The absorption bands, indicated with black arrows, of disulfide bridge bonds at 425 cm^−1^ and amine groups of proteins at 1600 cm^−1^ and 3400 cm^−1^ can be observed.

Additionally, TEM confirmed the adsorption of CTX onto the SiNPs’ surfaces (Figure 4a,b), and the FT-IR spectra revealed disulfide bridge bonds (R–S–S–R′) with absorption bands at 450 cm^−1^ (characteristic of CTX, as previously described by Aird et al. [37]) and amine groups in proteins (bands between 1600–1700 cm^−1^ and 3200–3500 cm^−1^) (Figure 4c).

**Figure 5:**
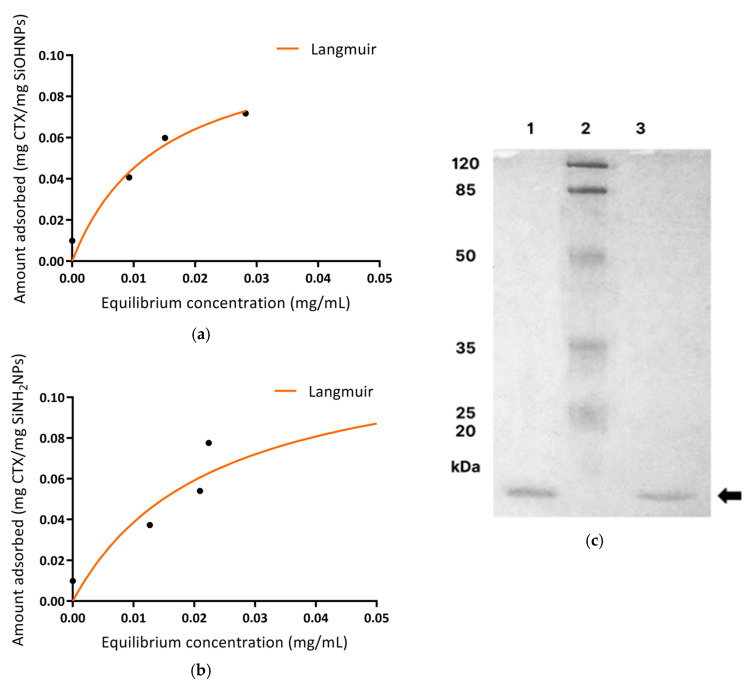
Adsorption isotherms. Isotherms of NV− (**a**) and NV+ (**b**) with different CTX concentrations (0.010, 0.050, 0.075, 0.1, 0.2). Both graphics adjust to the Langmuir model. Maximum adsorption capacity = 0.110 mg/mg SiOHNPs, R^2^ = 0.9452, and K_m_ = 0.0143 mg/mL (**a**); maximum adsorption capacity = 0.127 mg/mg SiNH_2_NPs, R^2^ = 0.9142, and K_m_ = 0.0229 mg/mL (**b**). (**c**). SDS-PAGE: bands corresponding to CTX desorbed from NV+ (1) and NV− (3) can be observed. The black arrow indicates CTX, and the second column (2) corresponds to the molecular marker.

As shown in Figure 5a,b, both types of SiNP isotherms presented a good fit to the Langmuir model but not to the Freundlich model. For SiOHNPs, the maximum AC was 0.11 mg/mL of SiOHNPs, and the equilibrium concentration necessary to reach 50% of the maximum adsorption capacity (Km) was 0.01 mg/mL (R^2^ = 0.94). In the case of SiNH_2_NPs, the maximum AC was 0.13 mg/mL of SiNH_2_NPs, and Km = 0.02 mg/mL with an R^2^ lower than the previous case (0.91).

Prominent bands corresponding to CTX (15 kDa) were observed in the desorbed NVs, the whole *C.d.t.* venom, and the isolated CTX by SDS-PAGE (14%) (Figure 5c). In the venom sample, additional bands corresponding to LAAO (L-amino acid oxidase, 50–70 kDa) [38] and CTM (5 kDa) were also detected, which are typically present in some *C.d.t*. specimens.

### 3.4. PLA_2_ Activity of CTX

The biological activity of isolated CTX and the synthesized NVs was tested by an indirect hemolytic assay [34]. Various samples (venom, CTX, NV+/−, and both types of bare SiNPs) generated hemolysis halos, expressed as a percentage with respect to the venom as a positive control (venom, 100% hemolysis). CTX exhibited the highest activity among the treatments (94.4 ± 5.55%), followed by NV+ (42.6 ± 3.18%) and NV− (41.6 ± 3.89%), both showing significant differences (*p* < 0.05) compared to CTX and the positive control. NVs retained the phospholipase activity characteristic of CTX, indicating that the biological activity of this enzyme was preserved. Neither type of bare SiNP exhibited hemolytic activity.

### 3.5. Cell Proliferation Assay (MTT)

**Figure 6:**
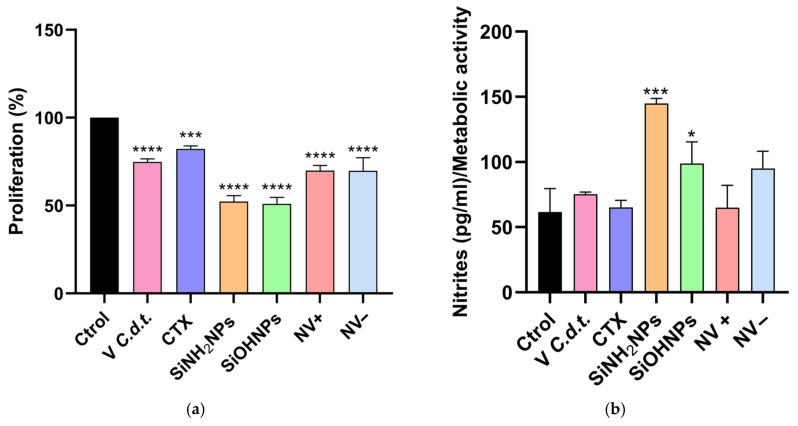
(**a**) Cell proliferation assay (MTT): cell proliferation (%) assay using THP-1 cell line. (**b**)**.** Nitrites: nitrite levels in the SN of the different treatments with respect to the metabolic activity. Asterisks indicate significant differences between treatments (* *p* > 0.05;; *** *p* > 0.001; **** *p* > 0.0001, *n* = 3).

THP-1 cells were exposed for 24 h to whole venom (positive control), CTX, SiNH_2_NPs, SiOHNPs, NV+, and NV– to evaluate their effects on cellular proliferation by the MTT assay (Figure 6a). All treatments induced a significant decrease in metabolic cellular activity with respect to the controls. Cells treated with venom showed similar metabolic activity (cytotoxicity) to that of isolated CTX (74.9 ± 1.70% vs. 82.2 ± 1.84%) and both NVs. On the other hand, NVs were demonstrated to be less cytotoxic than both types of SiNPs (69.9 ± 2.87% NV+ and 69.7 ± 7.52% NV− vs. 52.3 ± 3.41% SiNH_2_NPs and 51.0 ± 3.72% SiOHNPs), the latter being the ones that most affected cell proliferation, showing statistical differences compared to the other treatments.

Nitrite levels were also measured in the SNs (Figure 6b), as indicators of macrophage activation. These results were normalized to the MTT assay data, revealing an increase in nitrite levels for all treatments except NV+. SiNH_2_NPs and SiOHNPs were the only treatments that showed significant differences.

### 3.6. Cytokine and Nitrite Responses to SiNP and NV Treatments

**Figure 7:**
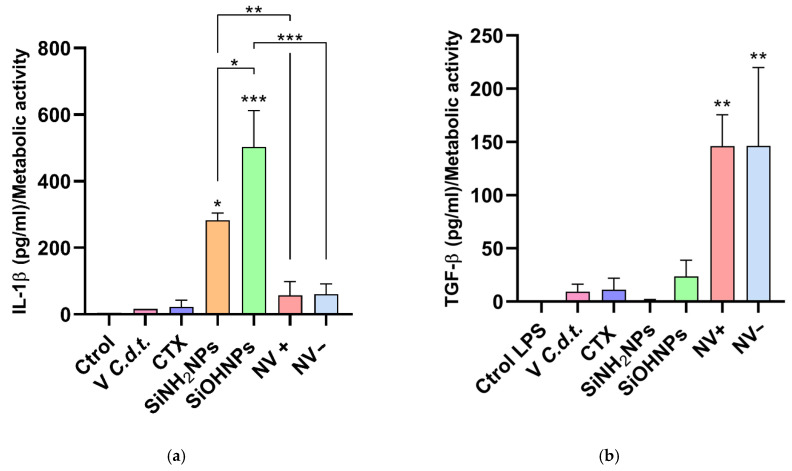
(**a**) IL-1β: ELISA of the cell SN of the different treatments. (**b**). TGF-β: ELISA of the cell SN after the LPS activation of the different treatments. Asterisks indicate significant differences between treatments (* *p* > 0.05; ** *p* > 0.01; *** *p* > 0.001, *n* = 3).

Pro-inflammatory (IL-1β) and anti-inflammatory (IL-10 and TGF-β) cytokines were measured by ELISA in the SNs of THP-1 cells after exposure to different treatments (whole venom, CTX, SiNH_2_NPs, SiOHNPs, NV+, and NV−) (Figure 7a,b), and results were normalized to the MTT assay data. For IL-1β, both types of NPs showed significant differences (*p* < 0.05) compared to the control, with SiOHNPs showing the highest concentration (503 ± 110), followed by SiNH_2_NPs (283 ± 21.5). In contrast, NV treatments did not show significant differences compared to the control group and had lower values than the SiNP treatments (NV− 61.2 ± 30.3 and NV+ 57.4 ± 40.7). The whole venom and CTX concentrations were lower than the control group (venom 16.5 ± 0.03, CTX 23.1 ± 19), which is expected since *C.d.t*. venom is not typically associated with a pro-inflammatory response. For IL-10 and TGF-β, none of the treatments showed significant differences with respect to the controls. To assess these cytokines’ secretion under a pro-inflammatory stimulus, THP-1 cells were activated with LPS (50 ng/mL). The results for TGF-β showed a significant increase in cytokine levels for both NV types compared to the control (146 ± 20.7 for NV+ and 146 ± 42.3 for NV−). In the venom and CTX groups, cytokine levels increased slightly, but no significant differences were observed (9.17 ± 4.16 and 11.1 ± 6.23, respectively). SiNPs also did not show significant differences (0.81 ± 0.80 SiNH_2_NPs and 23.7 ± 10.6 SiOHNPs). For IL-10, no significant differences were observed for any treatment.

## 4. Discussion

Ophidism is a worldwide issue that cannot be overlooked. In Argentina, most snakebite incidents are caused by snakes of the *Bothrops* and *Crotalus* genera [8]. Among the latter, *C.d.t.* is one of the leading causes of ophidian accidents due to its high lethality. After the snakebite, some of the venom toxins exert their immunomodulatory action by inducing leukocytes (mainly neutrophils and macrophages) to release cytokines, which are key mediators of inflammation. During envenomation, some of these toxins can cause tissue damage and release damage-associated molecular patterns (DAMPs) that are recognized by local cells, triggering an inflammatory response. In contrast, other venom components can exert the opposite effect and potentially downregulate inflammation [39].

Because of this, having antivenoms (AVs) available for treating snakebite incidents is of great importance to public health. In this sense, the use of NPs has generated significant interest in the biomedical field due to their ability to store or adsorb different types of drugs and proteins (for drug delivery or vaccine development) [26,28]. Many studies have tested the ability of these nanomaterials to adsorb or encapsulate different types of venom for AV production [40,41,42,43]. In this work, purified CTX from *C.d.t.* venom was adsorbed onto Stöber SiNPs of both charges (SiOHNPs and SiNH_2_NPs), generating complexes called NVs. Their physicochemical and biological activities, as well as their immunomodulatory properties, were studied to assess their potential as ADJs for the development of new AVs.

Our results also demonstrated the homogeneous synthesis of Stöber NPs for those treated with APTES, as well as spherical morphology, as expected for this method [31]. Moreover, the negative and positive net charges of both NP groups are consistent with their chemical groups. On the other hand, we obtained highly pure CTX from the whole *C.d.t*. venom, consistent with the high PLA_2_ content in this venom [13]. Regarding NV synthesis, 59% of the total CTX was adsorbed onto the SiNH_2_NPs surface (demonstrating 59% AC) and 48% onto the SiOHNPs. These results differ from those reported by Baudou [44], where higher venom detachment (15% in SiOHNPs and 20% in SiNH_2_NPs) was observed after 30 min. In our study, detachment after 30 min was only 2% for both NV+ and NV−. These differences may be attributed to the smaller SiNP size used in our study (150 nm vs. 400 nm in Baudou’s work) and the nature of the adsorbed proteins.

Proper CTX adsorption to both types of SiNPs was confirmed by TEM and FT-IR, showing characteristic absorption peaks. Adsorption isotherms were analyzed using Langmuir and Freundlich models. These models explain different forms of adsorption; Langmuir assumes that the number of sites on SiNPs’ surfaces where proteins can be immobilized is finite. Therefore, there is a maximum adsorption capacity, which results in the formation of a protein monolayer. On the other hand, the Freundlich model assumes the formation of multiple layers due to a heterogeneous surface with regions of high and low affinity [33,45,46]. Despite this, a progressive reduction in adsorption affinity still exists as a consequence of the repulsion generated by the adsorbed molecules. From the Langmuir model, we can obtain the maximum adsorption capacity as well as K_m_ (which represents the equilibrium concentration necessary to reach 50% of the maximum adsorption). From the Freundlich model, we can obtain K_f_ (adsorption capacity) and *n* values (adsorption intensity). In this work, in both cases, Langmuir was the model that better adjusted, indicating correct adsorption of the protein, which formed a monolayer over the surface of the SiNPs, with SiOHNPs presenting a greater adjustment with a higher R^2^ (0.94) than SiNH_2_NPs (0.91).

The presence of CTX in both types of NVs was corroborated as well, where the bands corresponding to this protein indicated the desorbed CTX from the negative and positive NVs. Moreover, we also demonstrated and corroborated that the CTX was successfully isolated from the whole venom, obtaining a single band by SDS-PAGE.

Then, we assessed the biological activity of the synthesized NVs using the THP-1 cell line. The cell proliferation assays (MTT) revealed that the NVs were shown to be more cytotoxic than the whole venom and CTX. In comparison, our results showed that the cytotoxicity of the NVs was higher than that obtained by Baudou et al. [44]. However, these results may differ as the whole venom was adsorbed onto the NPs, resulting in greater cytotoxicity due to the other components present. Although, in this work, the highest cytotoxicity was generated by SiNPs, it should be noted that the concentration of these was higher (0.60 mg) than NVs (0.17 mg SiNH_2_NPs and 0.20 mg SiOHNPs). This is because the concentration of NVs used in this assay was normalized based on the adsorbed CTX and not on the concentrations of SiNPs. In turn, the results of Baudou et al. show that SiOHNPs are much more cytotoxic, with a proliferation rate of approximately 20%, maybe due to the size of the SiNPs used (400 nm approximately). As De Marzi [47] demonstrated, the size of the NPs influences cell proliferation, with larger ones causing greater cytotoxicity. In the present work, it was observed that CTX presented lower cytotoxicity than *C.d.t* venom. In addition, the nitrite assay showed that SiNH_2_NPs treatment of THP-1 stimulated the highest release of this molecule and showed the highest value, which is expected because these SiNPs are highly immunogenic [28]. These results are consistent with those of previous assays, where treatments with a high immune response involved SiNPs.

In previous works, we have shown that SiNPs have different effects on monocytes depending on the charge present on their surface [48]. However, when a protein corona forms on their surface, the effects of the charge disappear, so NVs behave similarly due to the protein’s protective effect on the charge. Therefore, it would be advisable to continue working with NVs that exhibit the highest CTX adsorption capacity.

Finally, pro-inflammatory IL-1β and anti-inflammatory IL-10 and TGF-β cytokines were measured by ELISA. SiOHNPs induced the highest IL-1β levels, followed by SiNH_2_NPs, consistent with their immunogenic properties [47]. In contrast, the venom of *C.d.t.* is known to generate anti-inflammatory action by inhibiting the spread and phagocytic activity of macrophages and neutrophils [39]. Hence, it is reasonable to observe higher Il-1β levels in SiNPs than in venom. NVs did not show significant differences either, but compared with CTX and the whole venom, the concentration of IL-1β was higher, perhaps attributable to NPs. THP-1 cells were treated with LPS in order to activate them, and IL-10 and TGF-β cytokines were measured again. Regarding TGF- β, both NVs were the only treatments to show significant differences compared to the LPS control. The concentration of these treatments was higher than those of the whole venom and CTX, which are known to generate an anti-inflammatory response. This shows that these complexes generate a greater immunogenic anti-inflammatory response compared to the other groups. In the case of IL-10, no significant differences were observed in any treatment. As reported by Alves et al. [49], CTX has demonstrated in in vivo studies the ability to inhibit the production of certain cytokines, including IL-10.

## 5. Conclusions

In short, NVs exhibit anti-inflammatory activity, probably due to CTX characteristics, nullifying the pro-inflammatory effect of SiNPs. Consequently, for the generation of AVs, NVs could be combined with SiNPs to enhance the immune response, leveraging the pro-inflammatory activity of SiNPs and the antigen presentation capabilities of NVs. This anti-inflammatory characteristic of NVs could be applied not only to the production of antivenoms but also to the development of therapies for various diseases.

In this work, NVs were synthesized from solid SiNPs and CTX isolated from *C.d.t.* venom, preserving their integrity and enzymatic activity. These NVs show potential as ADJs for AV production, since NVs could be used together with bare SiNPs, to increase immunogenicity with the latter and thus facilitate the presentation of the antigen (CTX) with NVs. This strategy could improve the immune response and lead to a higher titer of specific neutralizing antibodies for the generation of AVs. Therefore, SiNPs offer promising tools for developing AVs and new therapies for various human diseases.

## Data Availability

The data can be shared up on request.

## Abbreviations

The following abbreviations are used in this manuscript:

*C.d.t.*	*Crotalus durissus terrificus*
LAAO	L-amino acid oxidase
PLA2	Phospholipase A2
CTX	Crotoxin
TLE	Thrombin-like enzyme
CVX	Convulxin
CTM	Crotamine
AV	Antivenom
ADJ	Adjuvant
NP	Nanoparticle
SiNP	Silica nanoparticle
NV	Nanovenom
V	Venom
SN	Supernatant
FPLC	Fast protein liquid chromatography
PBS	Phosphate-buffered saline
TEOS	Tetraethyl orthosilicate
ON	Overnight
RT	Room temperature
APTES	3-aminopropyltriethoxysilane
FT-IR	Fourier-transform infrared spectroscopy
AC	Adsorbed capacity
LC	Load capacity
TEM	Transmission electron microscope
DLS	Dynamic light scattering
MTT	Tetrazolium assay
ELISA	Enzyme-linked immunosorbent assay
